# Burned forests impact water supplies

**DOI:** 10.1038/s41467-018-03735-6

**Published:** 2018-04-10

**Authors:** Dennis W. Hallema, Ge Sun, Peter V. Caldwell, Steven P. Norman, Erika C. Cohen, Yongqiang Liu, Kevin D. Bladon, Steven G. McNulty

**Affiliations:** 1 Eastern Forest Environmental Threat Assessment Center, U.S. Department of Agriculture Forest Service, Southern Research Station, 920 Main Campus Drive, Venture Center II, Suite 300, Raleigh, NC 27606 USA; 2 Oak Ridge Institute for Science and Education, U.S. Department of Energy, 100 ORAU Way, Oak Ridge, TN 37830 USA; 3 Coweeta Hydrologic Laboratory, U.S. Department of Agriculture Forest Service, Southern Research Station, 3160 Coweeta Lab Rd, Otto, NC 28763 USA; 4 Eastern Forest Environmental Threat Assessment Center, U.S. Department of Agriculture Forest Service, Southern Research Station, 200 W.T. Weaver Blvd, Asheville, NC 28804 USA; 5 Center for Forest Disturbance Science, U.S. Department of Agriculture Forest Service, Southern Research Station, 320 Green Street, Athens, GA 30602 USA; 6 Department of Forest Engineering, Resources and Management, Oregon State University, 265 Peavy Hall, 3100 SW Jefferson Way, Corvallis, OR 97331 USA

## Abstract

Wildland fire impacts on surface freshwater resources have not previously been measured, nor factored into regional water management strategies. But, large wildland fires are increasing and raise concerns about fire impacts on potable water. Here we synthesize long-term records of wildland fire, climate, and river flow for 168 locations across the United States. We show that annual river flow changed in 32 locations, where more than 19% of the basin area was burned. Wildland fires enhanced annual river flow in the western regions with a warm temperate or humid continental climate. Wildland fires increased annual river flow most in the semi-arid Lower Colorado region, in spite of frequent droughts in this region. In contrast, prescribed burns in the subtropical Southeast did not significantly alter river flow. These extremely variable outcomes offer new insights into the potential role of wildfire and prescribed fire in regional water resource management, under a changing climate.

## Abstract (web summary)

Wildland fire seasons in the United States are getting longer, yet the impacts of fire on water availability at the regional scale are unclear. Here the authors show that fire increased annual river flow throughout the West, while prescribed burns in the subtropical Southeast had limited impact on river flow.

## Introduction

Wildfire seasons in the US are becoming longer due to recurring drought, coupled with more ignition sources and available fuel. However, it is unclear how this fire pattern affects water availability at the regional or continental scale^1–4^. Headwater forests supply more than 50% of water consumed in the contiguous US (CONUS)^5^ via streams and rivers—these forests are now susceptible to wildland fire risk or will become so in the near future^6,7^. River flow generally increases in the months following wildland fires^8^, but recent studies in New Mexico and Colorado also show that increased river flow can be sustained over multiple years^3,9^. Research in southern California suggests that increased post-fire river flow can potentially serve as an additional water resource during times of water scarcity^10^. Nonetheless, patterns and trends of post-fire river flow are expected to vary considerably between CONUS regions, depending on fire characteristics, hydroclimate, forest cover, and topography^11,12^.

The regional variability of hydrological responses to fire has major implications for forest management policies that aim to reduce wildland fire risk and sustain clean and abundant water supplies under a changing climate^13,14^. There is increased concern about the impact of fire on potable local water supplies^15,16^, resulting in costly challenges for municipal water production in the US^17^. The impacts of wildland fire on water quality and quantity at the regional scale are poorly understood^18^, however, we need such information for designing national resource management strategies that help reduce the wildland fire risk to water supplies.

In this study, we assess historical wildland fire impacts on river flow across the CONUS over the past 30 years. This first national empirical assessment includes rivers draining watershed areas between 10 and 100,000 km^2^, and compares wildland fire impacts on water for the entire spectrum of fire severity (prescribed burns to mega-fires), and across a broad range of climate and topography. We show that of the 168 locations studied, wildland fires affected annual river flow in 32 locations, where more than 19% of the basin area was burned. Wildland fires enhanced annual river flow in large parts of the Pacific Northwest, and even more so in the semi-arid Lower Colorado region, in spite of droughts experienced in this region. In southern and central California the enhancing impact of wildfire on river flow was masked by stronger, but opposing trends in precipitation, resulting in a net decline in river flow. In contrast, prescribed burns in the subtropical Southeast did not significantly alter river flow. The ensemble of post-fire river flow responses between and within regional basins, offers a new understanding of the potential role of wildfire and prescribed fire in water resource management.

## Results

### Observed changes in annual river flow

Our analysis of flow records from 168 CONUS rivers shows that observed changes in 5-year post-fire river flow coincided with local changes in precipitation and burn patterns (Supplementary Table 1). River flow in watersheds burned for at least 1% of their drainage area (burned area ratio, or BAR ≥ 1%), generally declined in nine water resource regions. These include the Mid-Atlantic (with a Mediterranean, or warm temperate Cfa climate in the Köppen classification system^19^), Tennessee (warm temperate Cfa, Cfb), Great Basin (arid BSk, continental Dsb and Dfb), South Atlantic-Gulf (Cfa), California (Csa, Csb), Great Lakes (Dfb), Rio Grande (BSk, Dfb), Texas-Gulf (Cfa) and Missouri (BSk, Dfa). In contrast, river flow predominantly increased in six other regions, viz., Lower Mississippi (Cfa), Upper Colorado (Dfb), Lower Colorado (BSk), Ohio (Cfa), Pacific Northwest (Csb, Dsb, Dfb), and Arkansas-White-Red River (BSk, Cfa, Dfa).

For the CONUS as a whole, river flow declined (median −5.9 mm or −5.7%) in fire-affected locations, in accordance with a negative precipitation trend (median −23.1 mm or −2.3%) (Supplementary Table 1). The greatest decrease in flow was observed in burned watersheds in the Great Basin (median of −45.4 mm or −37.1%), Rio Grande (−15.6 mm or −29.8%), Texas-Gulf (−10.7 mm or −25.4%) and California (−38.4 mm or −18.4%) regions. The greatest decrease in precipitation was also recorded for the Rio Grande (−100 mm or −13.1%), California (−35.4 mm or −4.6%), and Mid Atlantic (−59.5 mm or −4.3%) regions. Flow increased most in the Lower Mississippi (median of +160.1 mm or +27.4%), Lower Colorado (+9.9 mm or +25.6%) and Upper Colorado (+77.5 mm or +19.8%) regions. Likewise, the greatest increase in precipitation occurred in the Lower Mississippi (+311.2 mm or +23.3%) and Upper Colorado (+41.9 mm or +6.9%) regions.

However, there are burned watersheds where river flow increased notwithstanding a declining trend in precipitation. This was the common response in the Lower Colorado region (river flow +9.9 mm or +25.6% vs. precipitation −30.6 mm or −6.0%), where wildfires typically burned 15.4% of the watershed area, and to a smaller extent in the Pacific Northwest region.

### Wildland fire impacts on annual river flow

**Fig. 1:**
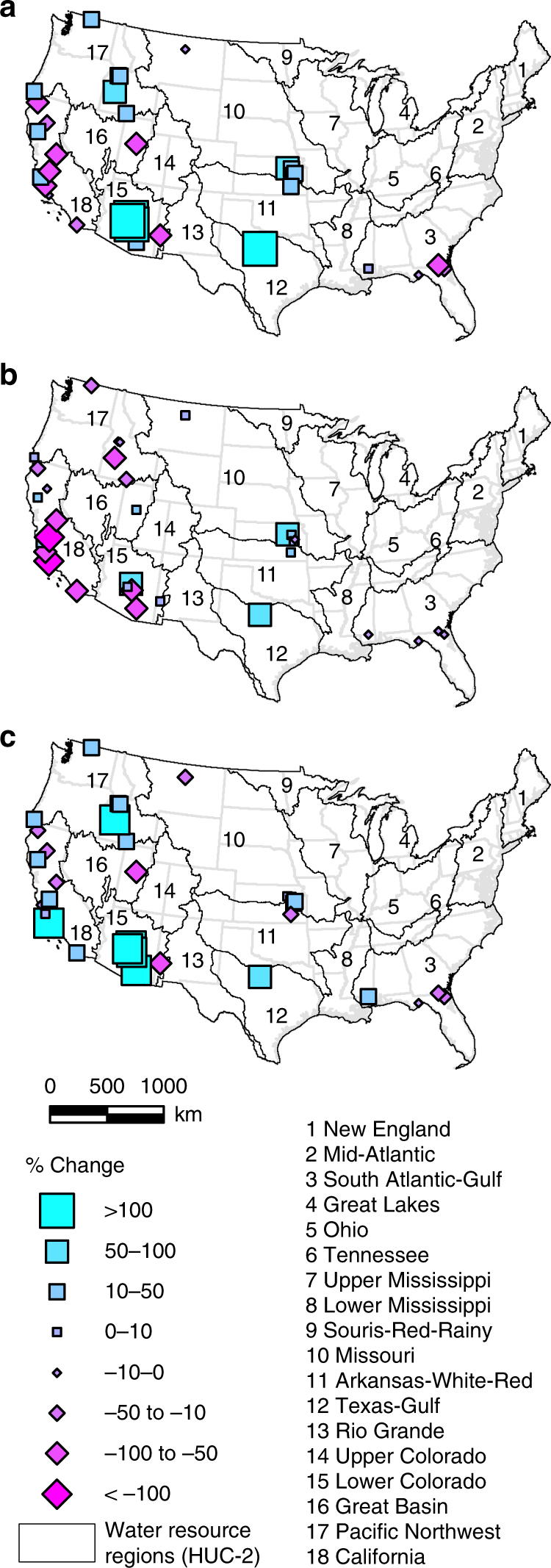
Change of annual river flow in US watersheds burned at any time between 1985 and 2008. Percentage observed change in 5-year mean annual river flow (**a**), percentage change attributed to climate (**b**), and percentage attributed to wildland fire (**c**). Attribution was performed using climate elasticity models fitted for each watershed individually (*n* = 32 burned watersheds with BAR ≥ 19%)

**Fig. 2:**
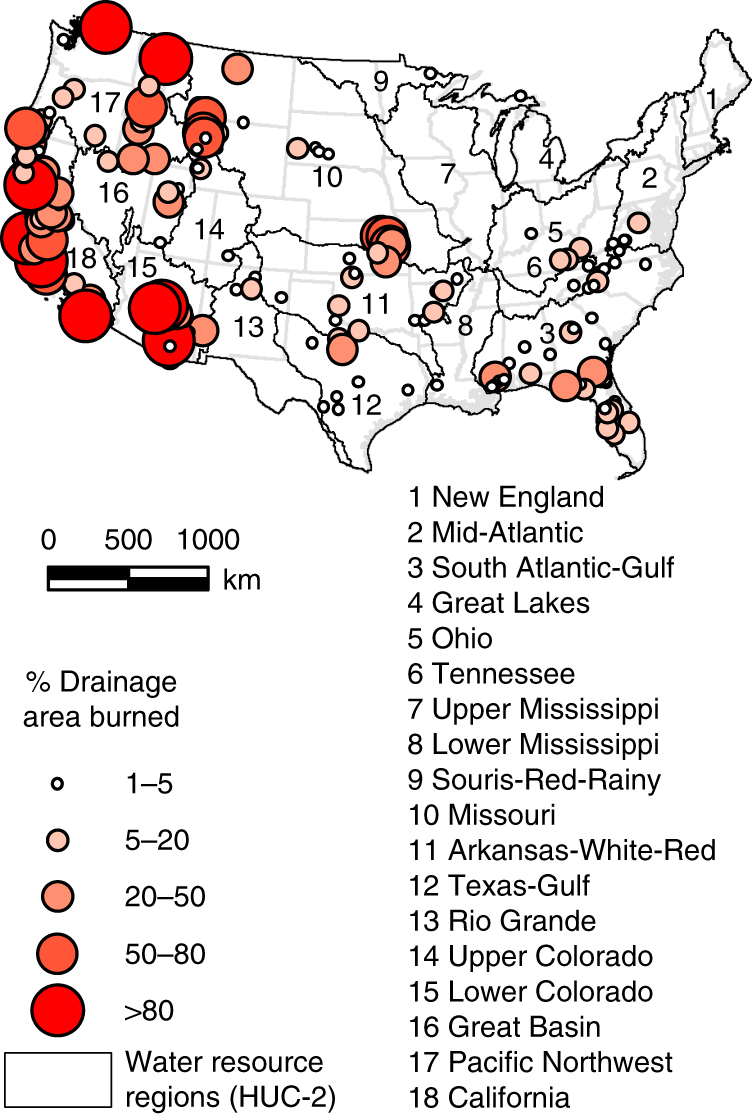
Ratios of watershed area burned to watershed drainage area. Shown are US watersheds burned between 1985 and 2008 (*n* = 168 burned watersheds with BAR ≥ 1%)

Large wildland fires enhanced annual river flow for at least 5 years, even in areas affected by recurring drought (Fig. 1). This was especially the case throughout the Pacific Northwest, where fires occurred in areas with a Mediterranean or humid continental climate, and in the semi-arid Lower Colorado region where wildfires burned large portions of headwater catchments (Fig. 2). Similarly, wildland fire increased the annual river flow in the humid subtropical Texas-Gulf region, and in Mississippi, where rapid vegetation growth produces massive amounts of fuels susceptible to fire.

Fire effects on river flow were not always immediately evident in the recorded flow data, due to severe weather events (e.g., post-burn drought or flooding) and variations in the short term (5-year) climate that offset or masked the fire impacts. Therefore, we isolated the wildland fire impact on river flow, by subtracting the local climate-based flow prediction for each fire^12,20^ (Fig. 1c cf. Fig. 1a, b). We detected the largest fire-induced increase in river flow in the Lower Colorado, Pacific Northwest, and Texas-Gulf regions. In southern California and the Pacific Northwest, in particular, increases in river flow corresponded with a high severity of wildland fires.

Large watersheds in the drought-prone Western US experienced the largest uncontrolled fires. High wind speeds and low humidity levels in the lower atmosphere and in vegetation, allow fires to spread rapidly, burning large portions of watersheds in California (median BAR 20.6%), the Lower Colorado region (15.4%), and the Pacific Northwest (13.5%) (Fig. 2 and Supplementary Table 1). Fire impacts on river flow were most evident in four Arizona rivers (Lower Colorado) with a high BAR (>39%), increasing flow by more than +128% (Fig. 1c). In the Pacific Northwest and Texas-Gulf regions, flow increased by +24% (median of 6 rivers) and +98% (1 river), respectively. In many other rivers with upstream areas affected by a fire, changes in annual river flow associated with wildland fires were less than +15% (median for CONUS). The smaller river systems east of the Mississippi River (52 rivers) had the lowest BAR, typically below 5% (Fig. 2). This is less than the BAR for rivers in the Western US (median of +9.6%, 116 rivers).

Not all wildland fires affected annual river flow. For example, prescribed (controlled) burning, conducted to reduce accumulated fuels such as dead vegetation and fallen branches, had only a limited effect in the subtropical Southeast. Prescribed burns are generally conducted during favorable weather and vegetation conditions. The severity of the prescribed burns was limited, and their size below a critical minimum (BAR_t_ = 19%) needed to affect river flow. This was notably the case in relatively large, low-altitude (<500 m) watersheds like those in the Texas-Gulf region (BAR = 2.3%), and in the South Atlantic-Gulf Region (3.1%), where smaller prescribed burns are more common than elsewhere in the US.

### Climate and other factors influencing river flow response

**Fig. 3:**
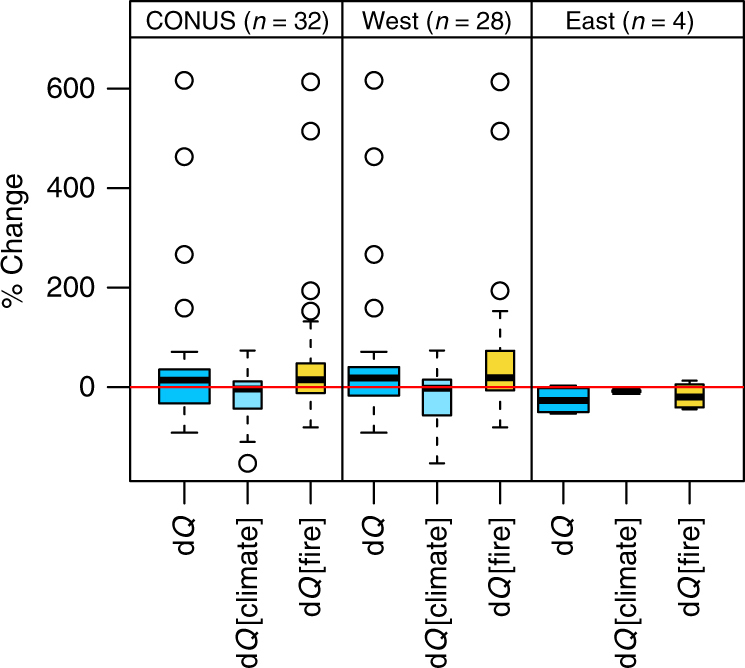
CONUS-East-west comparison of post-fire change in annual river flow. Percentage change in 5-year mean annual river flow observed (d*Q*) attributed to climate (d*Q*[climate]) and fire (d*Q* [fire]), respectively, for watersheds burned between 1985 and 2008 (BAR ≥ 19%). Results are summarized for the CONUS (*n* = 32) and for the regions west (*n* = 28) and east (*n* = 4) of the Mississippi River, respectively. Whiskers extend to the most extreme value no more than 1.5× interquartile range from the box

General patterns of post-fire river flow were not only caused by wildland fire, but also followed the interannual variability in climate conditions (Fig. 3). For example, in northern California (Csb climate type), river flow declined post-fire mainly as a result of short-term drought (Fig. 1a cf. Fig. 1b). With some exceptions, wildland fire and climate contributions to flow increase in the lower Missouri region were comparable (<10%; Dfa). Conversely, wildland fire impacts were greater in southern and central California (>50%; Csa, Csb; Fig. 1c), but declining precipitation trends masked the enhancing impact of wildfire on river flow. These opposite trends resulted in a net decline in river flow. We detected the most extreme responses in mid-elevation parts of the Pacific Northwest (Dsb) and Lower Colorado (BSk) regions, where wildland fire increased flow >100% (Fig. 1c).

One-half of the fires affected less than 6% of the area of a gaged watershed. For the ensemble of watersheds in the CONUS affected by a fire of any given size or extent (BAR ≥ 1%), precipitation characteristics had the greatest influence on 5-year mean annual river flow (Supplementary Fig. 1a). Changes in annual precipitation and monthly precipitation variance had influences of >47% and >4.9%, respectively, of the total impact on river flow. The relative influence of the change in monthly precipitation variance was greater for the West (9.5%) compared to the East (4.9%) (Supplementary Fig. 1b cf. Supplementary Fig. 1c). Furthermore, absolute values of precipitation (which includes snowfall) (2.4%) also had a greater influence. The greater overall importance of precipitation variables in these Western watersheds with higher elevation and steeper topography is explained by a more extreme climate characterized by higher interannual and seasonal variability.

By contrast, in the Eastern CONUS, the proportion of the watershed with unvegetated land cover (barren land and urban area) had some influence on river flow (4.1% and 2.3%, respectively), secondary to climate factors (51% and 4.9% for annual precipitation and monthly precipitation variance, respectively) (Supplementary Fig. 1b cf. Supplementary Fig. 1c). At the CONUS scale, river flow was mostly influenced by precipitation change (47.4%), change in monthly precipitation variance (8.3%), pre-fire monthly precipitation variance (4.5%) and post-fire monthly precipitation variance (3.3%) (Supplementary Fig. 1a). Given the small size of the vast majority of wildland fires compared to the drainage area of affected watershed, the influence of fire on river flow in the CONUS (<1%) was typically overshadowed by climate, topography and land cover.

A different picture emerges when examining the fires that affected a larger portion of a watershed. Wildland fire that burned 19% or more of the watershed area generally increased river flow. This impact was related to the proportion of the watershed affected by moderate-to-high burn severity (relative influences 2.2% and 2.8%, respectively) for a minimum of 3.8% and 6.5%, respectively (Supplementary Fig. 2). Areas affected by high severity burning had a consistently greater influence on river flow change (up to 4.0% for BAR ≥ 25%) than areas affected by moderate burning. Areas with low severity burning impacts were too small and their influence on river flow fell below that of a random variable introduced into the analysis for comparison, even for BAR ≥ 25%. Despite the uncertainty in burn severity classification thresholds, especially for moderate and high burn severity classes^21^, we report a consistently greater influence on river flow when these classes were accounted for individually.

### Implications for post-fire forest and water management

Our study suggests that both climatic variability and fire characteristics affect river flow. It is not sufficient to focus on post-wildland fire management strategies for mitigating hydrological impacts associated with flooding and erosion. Post-fire management strategies also need to be flexible and adaptive, locally and regionally. However, the post-fire hydrological impacts often depend on chance post-fire weather events. Regions that have not yet experienced any large post-fire hydrological impacts (e.g., floods) because of drought, may suffer from catastrophic disaster at a later time when precipitation increases again. This creates major challenges to anticipate and manage.

The outcomes are consistent with existing theories, suggesting that multi-year (i.e., <10 years) increases in post-fire water yield can increase municipal water supplies during times of water scarcity^4^. Our results corroborate with recent evidence of significant increases in river flow following wildland fires in arid watersheds, such as New Mexico and Colorado^9,16,22^. Interestingly, the greater portion (65.5%) of the upstream burned area for rivers with a notable increase in river flow (>10%) in the Lower Colorado, Pacific Northwest and Texas-Gulf regions, was characterized by low burn severity. However, the likely reason that prescribed burning effects on annual river flow in the South Atlantic-Gulf region were not significant is that these burns were small compared to the size of the watersheds in which they were conducted—90% were burned across an area smaller than the critical relative burned area threshold BAR_t_ = 19%. Climate elasticity modeling was also complicated by severe tropical weather events (2006 and 2008 hurricane seasons^23,24^) in this region (Florida, Georgia, and Mississippi), and we believe that the outcomes of the attribution analysis for these areas do not reflect the actual fire impact. Findings invariably point to the relative size of a fire as a factor limiting impacts on water supply. But, the role of burn severity is more complex because burn patterns tend to alter the path along which precipitation is transferred to the river^25^. Burn impacts of moderate-to-high severity affected the river flow in watersheds with fires exceeding BAR_t_, mainly because vegetation mortality increases runoff. This explains why the critical relative burned area threshold we found (BAR_t_ = 19%), is very close to the 20% threshold traditionally reported in forest harvest studies^26^. Prescribed burns, on the other hand, are often designed to reduce canopy mortality, therefore more and larger fires must be examined to properly assess the potential role of prescribed fires as a water augmentation practice in headwater catchments. Such a potential, if validated regionally, would introduce a secondary use of prescribed burning, in addition to its traditional purpose of fuels load reduction.

This paper underscores the need for a better understanding of where water resources are most likely to be affected^4^ by wildland fire, given the large variability of post-fire river flow responses between and within regional basins. We believe that knowledge of these regional differences is critical in developing national strategies to respond to increasing wildland fires with limited resources. Reliable impact assessments will help determine where prescribed burning may be applied with minimal negative impacts on water supply. The accuracy of such assessments will become increasingly important, as global wildland fire hazards continue to increase with a changing climate, and a growing demand for water and water-related services.

## Methods

### Datasets

We retrieved high-resolution spatial datasets and time series for CONUS wildland fire, hydrology, climate, topography and land cover (Table 1). Wildland fire locations, dates, extent and burn severity were obtained from the Monitoring Trends in Burn Severity (MTBS) dataset^27^. MTBS is currently the best dataset available for the purpose of this analysis, because it contains the largest historical record of CONUS wildland fires larger than 405 ha (1000 acres) in the West, and larger than 202 ha (500 acres) in the East, respectively, between 1984 and 2016. Moreover, it was mapped at high resolution (30 × 30 m). This dataset reports five burn severity classes, viz. unburned or underburned, low severity, moderate severity, high severity and increased greenness. These classes were derived from the differenced normalized burn ratio (dNBR). dNBR in this product was calculated using bands 4 (near infrared) and 7 (mid-infrared) from Landsat Thematic Mapper (TM) and Enhanced Thematic Mapper (ETM+) images obtained before and after wildland fires. MTBS has been the dataset of choice in studies on CONUS-wide trends in burn severity and area^28,29^, forest disturbance^30^, and vegetation type conversion after wildland fire^31^.

**Table 1:** Characteristics of high-resolution spatial datasets and time series used to determine wildland fire impacts on river flow

Dataset	Description	Format	Resolution	Period	Version date	Source
MTBS burned area boundaries	Fire attributes	Spatial vector	–	1984–2014 Annual	9/25/2014	http://www.mtbs.gov
MTBS burn severity mosaic	Burn severity	Spatial raster	30 × 30 m	1984–2014 Annual	9/25/2014	http://www.mtbs.gov
GAGES-II	River flow	Time series	–	1980–2014 Daily	2016	10.5066/F7P55KJN
GAGES-II Geospatial attributes	Watershed boundaries	Spatial vector	–	2011	2016	10.5066/F7P55KJN
WBD watershed boundary dataset	HUC-2 Water resource regions	Spatial vector	–	–	2015	https://data.nal.usda.gov/dataset/watershed-boundary-dataset-wbd
Daymet v3	Climate	Spatial raster time series	1 × 1 km	1980–2014 Daily	9/30/2016	10.3334/ORNLDAAC/1328
PRISM	Climate	Spatial raster time series	4 × 4 km	1980–2014 Monthly	2013	http://www.prism.oregonstate.edu
GMTED2010	Elevation	Spatial raster	244 × 244 m	2010	2010	https://lta.cr.usgs.gov/GMTED2010
NLCD 2001	Land cover	Spatial raster	30 × 30 m	2001	2011	http://www.mrlc.gov/nlcd2011.php

Abbreviations: CONUS, contiguous United States; DAAC, Distributed Active Archive Center; EROS, Earth Resources Observation and Science; MTBS, GMTED, Global Multi-resolution Terrain Elevation Data; Monitoring Trends in Burn Severity; NASA, National Aeronautics and Space Administration; NLCD, National Land Cover Database; ORNL, Oak Ridge National Lab; PRISM, Parameter‐elevation Regressions on Independent Slopes Model; RSAC, Remote Sensing Applications Center; USDA, United States Department of Agriculture; USGS, United States Geologic Survey

Next, we collected watershed attributes (boundaries, drainage areas and perimeters) and daily time series of river flow from the GAGES-II dataset (Geospatial Attributes of Gages for Evaluating Streamflow, version II)^32^ (Table 1). The boundaries of the water resource regions were acquired from the Watershed Boundary Database^33^. We extracted climate data from the daily high resolution (1 × 1 km) Daymet v3 dataset^34^ and obtained the gridded PRISM (Parameter-elevation Regressions on Independent Slopes Model) dataset^35^. For topographic data, we used the highest resolution version (244 × 244 m) of the Global Multi-resolution Terrain Elevation Data 2010 (GMTED2010), principally obtained during the Shuttle Radar Topography Mission^36^. Finally, we obtained land cover from the 2001 National Land Cover Database (NLCD)^37^.

### Database of burned watersheds in the CONUS

GAGES-II reference watersheds (i.e., non-urban watersheds with minimum human disturbance) were filtered based on a drainage area >10 km^2^ and available river flow data, as documented in the GAGES-II metadata (Supplementary Fig. 3). Nested watersheds were also filtered. The watershed polygons and MTBS burn severity raster layers were then combined into a 120-m resolution grid, for each annual MTBS layer available for the period between 1984 and 2013. This yielded data layers documenting the unburned or underburned, low severity, moderate severity, high severity, and increased greenness areas within the burned watersheds.

The following step was to calculate the annual burned area to drainage area ratios (BAR). Watersheds burned for as little as 1% of their drainage area (BAR ≥ 1%) in any single year between 1984 and 2008 were included in the plenary set of burned watersheds. This plenary set served to identify the relative influence of fire and other environmental variables on river flow, and to detect the minimum threshold of area burned to drainage area (BAR_t_), above which fire affects river flow.

We also collected the fire dates for the plenary set of burned watersheds, and aggregated discharge (*Q*) and climate data (precipitation *P*, monthly precipitation variance $\sigma _{P_{\rm m}}^2$, PET, and the amount of water contained within the snowpack SWE) for the 5 years preceding and following wildland fire. Monthly potential evapotranspiration (PET) was estimated from PRISM data using Hamon’s method^38,39^:

1 $${\mathrm {PET}} = 29.8 \cdot {\rm Hr}_{\mathrm {day}}\frac{{e_{\mathrm {sat}}(T)}}{{(T + 273.2)}}$$

where PET is given in mm d^−1^, Hr_day_ is the number of daylight hours and *e*_sat_ is the saturation vapor pressure (kPa) for mean air temperature *T*.

Lastly, we aggregated land cover from NLCD and topographic attributes (elevation, slope, and aspect) from GTMTED2010. The Gravelius’ compactness factor *C* was determined to obtain an estimate of the compactness—or plan shape—of the watersheds^40,41^:

2 $$C = \frac{{Pm}}{{2\sqrt {\pi A} }}$$

where *C* equals the ratio of the watershed perimeter Pm to that of a circle with the same area *A*. A subset of more severely affected watersheds (BAR > BAR_t_), was used in the assessment of total fire impact on river flow.

### Assessment of wildland fire impacts on river flow

**Fig. 4:**
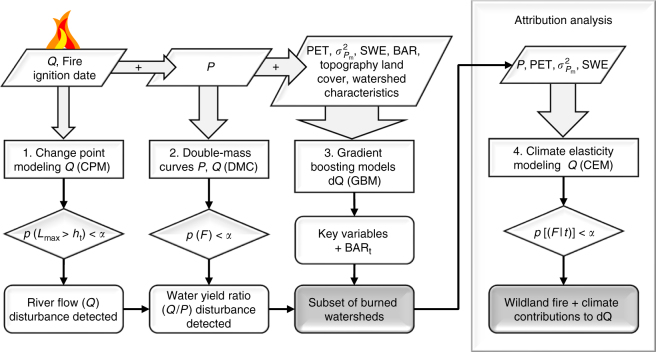
Framework for evaluating wildland fire impacts on river flow. *Q*: river flow, *P*: precipitation, $\sigma _{P_{\rm m}}^2$: monthly precipitation variance, SWE: snow water equivalent, PET: potential evapotranspiration, BAR: burned area to drainage area ratio, *L*_max_: maximized value of the Lepage statistic, *F*: *F*-statistic, *t*: *t*-statistic, *α*: significance level

We evaluated wildland fire impacts on river flow using a framework that we programmed in R^12,42^ (Fig. 4). Following a stepwise approach, we added progressively more data at each step, to highlight various aspects of post-fire river flow response and interactions with other parameters. In an initial assessment, we detected disturbance of river flow (*Q*) (step 1) and water yield ratios (*Q/P*) (step 2). Next, we evaluated the nonlinear relationships and interactions between BAR, watershed geometry, climate variability, topography and land cover, in order to determine their respective influences on d*Q* and the critical minimum value of BAR (i.e., BAR_t_) resulting in river flow disturbance (step 3). Finally, we separated the fire disturbance impact on river flow from the climate variability impact. This was done by means of attribution analysis of a subset of burned watersheds (*BAR* ≥ BAR_t_) for which a water yield ratio disturbance was detected (step 4).

River flow disturbance was determined by analyzing *Q* for the 5 years before and after wildland fire with the change point model (CPM) (Fig. 4). The null hypothesis was defined as no change in monthly *Q* in the year following wildland fire, written as:

3 $$H_0:Q_i\sim F_0(\theta _0),\forall i$$

where *Q* at any time step *i* follows distribution *F*_0_ estimated by parameter set *θ*_0_. This hypothesis was evaluated with the non-parametric Lepage statistic^43^. The Lepage statistic combines the Mann–Whitney statistic (or Wilcoxon rank-sum) for detecting location shifts (ranked values of *Q*), and the Mood statistic for detecting scale shifts (dispersion of *Q*):

4 $$L = U^2 + M^2$$

where *U* is the Mann–Whitney statistic and *M* the Mood statistic. The Mann–Whitney statistic is given by^44^:

5 $$U = {{\rm min}}\left\{ {U_S,U_T} \right\}$$

6 $$U_S = n_Sn_T + \frac{{n_S(n_S + 1)}}{2} - r(x_i)$$

7 $$U_T = n_Sn_T + \frac{{n_T(n_T + 1)}}{2} - r(x_i)$$

where *S* and *T* represent the sets of observations preceding and following a presumed change point *τ*, respectively, *n* is the corresponding number of observations, and *r*(*x*_*i*_) gives the pooled rank sums of all observations. The Mood statistic is defined as^45^:

8 $$M = \left| {\left( {M\prime - \mu _{M\prime }} \right)/\sigma _{M\prime }} \right|$$

with

9 $$M\prime = \mathop {\sum}\limits_{x_i{{\in }}S} {\left( {r(x_i) - (n + 1)/2} \right)^2}$$

10 $$\mu _{M\prime } = \frac{{n_S\left( {n^2 - 1} \right)}}{{12}}$$

11 $$\sigma _{M\prime }^2 = n_Sn_T(n + 1)(n^2 - 4){{\rm /}}180$$

where *μ*_*M′*_ and *σ*_*M′*_^2^ are the mean and variance of the Mood statistic, respectively. The Lepage test can detect general types of distribution change and is considered more powerful than comparable non-parametric tests for detecting changes in hydrological time series data^46^. We used the cpm package^47^ in R to run through each time series of *Q* and compare value distributions before (*F*_1_) and after (*F*_2_) each time step. A change was detected when *L* exceeded critical value *h*_t_ corresponding with significance level *α* = 0.05, in which case *F*_1_ ≠ *F*_2_^48^, and the best estimate of the timing of disturbance (i.e., the change point) corresponded to the maximized value of the test statistic (*L*_max_). The null hypothesis was rejected if this timing occurred within one year following the fire assuming that river flow disturbance was associated with the fire.

Disturbance in the water yield ratio (*Q*/*P*), i.e., the amount of river flow per unit of precipitation, was detected via double-mass analysis of *Q* and *P* for the same period (Fig. 4). The null hypothesis for this flow characteristic was defined as no change in monthly water yield ratio, or in formula:

12 $$H_0:Q_{{{\mathrm {cum,}i}}}\sim F_0(P_{{{\mathrm {cum,}i}}};\theta _0),\forall i$$

where cumulative river flow *Q*_cum_ follows a distribution *F*_0_ estimated by cumulative precipitation *P*_cum_ and parameter set *θ*_0_. This we evaluated by performing ordinary least square regression of the double mass relationship for the pre and post-fire periods separately (the restricted models), and combined (unrestricted model). Subsequently, we tested the equality of variances with Chow’s *F*-test. The *F*-statistic is defined as^49^:

13 $$F = \frac{{\left\{ {{\mathrm {SSE}}_0 - ({\mathrm {SSE}}_1 + {\mathrm {SSE}}_2)} \right\}/K}}{{({\mathrm {SSE}}_1 + {\mathrm {SSE}}_2)/(n - 2K)}}$$

where SSE_0_ is the sum of squared errors for the restricted linear model, fitted to the pooled data, and SSE_1_ and SSE_2_ are the sums of squared errors for the unrestricted linear models fitted to the respective subsets of the data. *n* equals the number of samples and *K* is the number of regressors. Whenever the *F*-statistic was significant, the evaluated fire date corresponded with a structural break in the double-mass curve (DMC), indicating a water yield disturbance.

Gradient boosting is a machine learning technique that builds regression trees of sample data in a sequential process, where a simple model (base learner) is fitted to pseudo-residuals at each iteration^50^. A loss function is minimized at each fold along the gradient defined by these pseudo-residuals, allowing the program to learn progressively more about relationships between the data. We used the gbm package^51^, which is an implementation in R of Friedman’s stochastic gradient boosting machine (GBM)^50^ based on Freund and Schapire’s AdaBoost algorithm^52^. This GBM increases robustness by selecting samples randomly, and has previously been employed in studies on *Q* trend analysis and prediction^53,54^. Here, we built a GBM of d*Q* accounting for nonlinear relationships and interactions between 49 variables describing BAR, watershed geometry, climate variability, topography and land cover, by minimizing the squared error (Fig. 4). We introduced two random variables to discern influential variables from non-influential variables. We allowed interaction depth *K* equal to the number of input variables (*K* = 49), resulting in a richer model in comparison to more compact trees (*K* ≈ 5). The learning rate was set to *λ* = 0.001, and the program terminated after a fixed number of iterations *M* = 30,000 (the total number of trees to fit). Finally, we estimated the relative influence *I*_*j*_ of each variable *x*_*j*_ on the variation of d*Q* as^50^:

14 $$\hat I_j^2 = \frac{1}{M}\mathop {\sum }\limits_{m = 1}^M \hat I_t^2(T_m)$$

where $\hat I_t^2$ is the empirical improvement in squared error obtained for the collection of decision trees $\left\{ {T_m} \right\}_1^M$ through boosting, averaged over a collection of *M* trees created before reaching the best performance founds by means of five-fold cross-validation. GBMs were constructed in this manner for the plenary set of burned watersheds (BAR ≥ 1%). The plenary set was sufficiently large to allow identification of environmental variables impacting d*Q* in the Eastern CONUS (*n* = 52) and Western CONUS (*n* = 110) individually. Subsequently, we built GBMs of watershed subsets, applying a variable lower limit for BAR equal to (1, 10, 15, 16, 17, 18, 19, 20 and 25%) to locate the critical threshold BAR_t_ affecting d*Q*.

Disturbance in *Q* or *Q/P* aside, the total impact of wildland fire on *Q* was determined with greater accuracy by filtering out more the complex climate variability effects established by the GBM (Fig. 4). This furthermore allowed us to account for the cases where climate variability potentially offset or enhanced *Q*. Climate elasticity models (CEMs) are especially suited for this purpose and commonly applied in the attribution of river flow disturbance^12,55^. CEMs with various combinations of the most influential climate parameters (*P*, PET, $\sigma _{P_{\rm m}}^2$ and SWE) were fitted to predict d*Q* based on changes in the 5-year mean annual climate (Supplementary Table 2).

The best CEM for each burned watershed was identified based on the lowest value of the Bayesian Information Criterion (BIC score) calculated as^56^:

15 $${\mathrm {BIC}} = - 2{{\rm ln}}(L_k) + k{{\rm ln}}(n)$$

with *L*_*k*_ the maximized likelihood, *k* the number of parameters in the model and *n* the sample size. The South Atlantic-Gulf, Missouri, Lower Colorado, Pacific Northwest and California regions combined had seven burned watersheds where a climate elasticity model of *Q* (CEM_4_) with parameters for d*P* and dSWE (change in snow water equivalent) yielded the best fit (Supplementary Fig. 4a), especially for watersheds located at higher elevations (typically >450 m) and with steep topography (Supplementary Fig. 4b). CEM_3_ with parameters for d*P* and ${\rm d}\sigma _{P_{\rm m}}^2$ (change in monthly precipitation variance) was the best model for most watersheds (*n* = 11). These predominantly humid watersheds (a lower quartile *P*_Q25_ > 656 mm) were located in most regions above 300 m. CEM_2_ with parameters for *P* and PET yielded the best fit for ten watersheds at lower elevation. The one-parameter (d*P*) CEM_1_ was the best model for four watersheds with the steepest terrain, although not necessarily at high elevation. Supplementary Fig. 5 summarizes the results of the attribution analysis per water resource region, discussed in the main paper. The significance of these CEMs was evaluated using the *F*-test (CEMs with multiple climate variables) and *t*-test (one-parameter CEMs with insufficient degrees of freedom to calculate the *F*-statistic).

### Hypothesis testing

CPMs, DMCs and CEMs were calculated for all burned watersheds in the plenary set (BAR ≥ 1%, *n* = 168), and discussed here for watersheds burned over an area exceeding the critical threshold for impact on river flow (BAR ≥ 19%, *n* = 43) (Supplementary Fig. 6). Indicators signaling that factors other than precipitation affected river flow (e.g., wildland fire) were found by comparing the outcomes of statistical significance testing on monthly *Q* and water yield ratios (d*Q/*d*P*) performed with the CPM and double-mass analysis (DMC), respectively. Subsequently, climate elasticity modeling (CEM) allowed us to estimate total climate impact on *Q*. d*Q* in the year following wildland fire was significant in 13 of 43 burned watersheds (all in the Western CONUS) (Supplementary Fig. 6a), however a change (breakpoint) in (d*Q/*d*P*) was detected in the same watersheds plus 23 additional watersheds (all in the Western CONUS) (Supplementary Fig. 6b c.f. Supplementary Fig. 6d). Finally, climate elasticity of d*Q* could be determined for six more watersheds (Supplementary Fig. 6c c.f. Supplementary Fig. 6d). The CEM was not significant for four burned watersheds (Supplementary Fig. 6d), because only pre-fire annual data was used, reducing the significance of the test statistic. The total number of burned watersheds with significant climate elasticity of river flow was 38, and we performed the attribution analysis for 32 of these for which a breakpoint was detected.

### Attribution analysis of river flow disturbance

Total impact of wildland fire on *Q* disturbance for burned watersheds was calculated as the difference between observed river flow disturbance (d*Q*) and expected d*Q* based on climate variability calculated with the CEM^12,57^:

16 $${{\rm \Delta }}Q_{{\rm dist}} = {{\rm \Delta }}Q_{\mathrm {obs}} - {{\rm \Delta }}Q_{\mathrm {clim}}$$

Wildland fire was presumed the principal cause of ${{\rm \Delta }}Q_{{\rm dist}}$ in watersheds where BAR ≥ BAR_t_, although some western watersheds may have been affected by additional disturbances not accounted for, e.g., beetle outbreaks.

### Code availability

Relevant code may be rendered available from the corresponding author upon reasonable request.

### Data availability

The datasets generated in this study are available from the corresponding author upon reasonable request.

## Electronic supplementary material

Supplementary Information(PDF 1135 kb)
