# Supplementary material for: Burned forests impact water supplies
Source: Nat Commun. 2018 Apr 10;9:1307. doi: 10.1038/s41467-018-03735-6 (PMC5893570; doi:10.1038/s41467-018-03735-6)
Supplement: Supplementary file 1 — Supplementary Information(PDF 1135 kb) [file 41467_2018_3735_MOESM1_ESM.pdf]

Supplementary Information

Burned forests impact water supplies

Hallema et al.

Supplementary figures

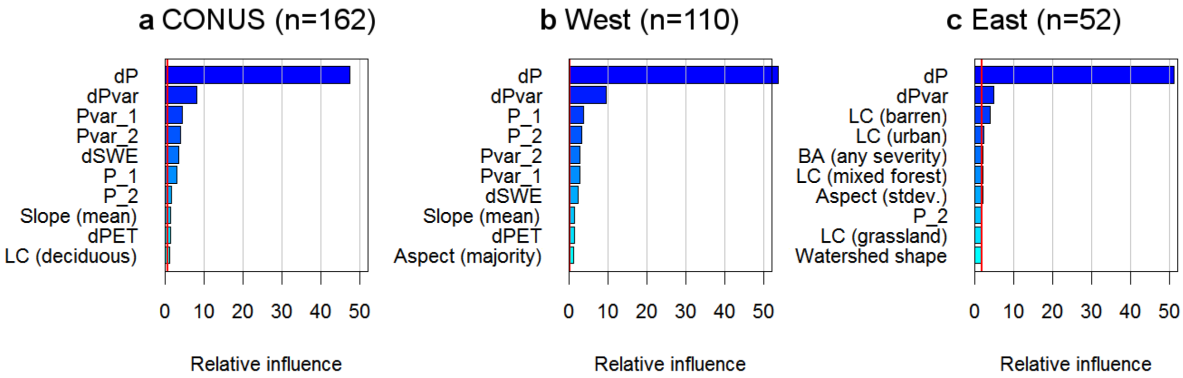

Supplementary Figure 1. CONUS-East-West comparison of variables affecting post-fire annual river flow. Results show relative influence of variables for the CONUS (**a**), and west (**b**) and east (**c**) of the Mississippi River. Influence is expressed of the percentage of total influence of all variables on 5-year post-fire mean annual river flow, and was calculated based on the improvement in performance of the boosted regression model after inclusion of displayed variables. The relative influence of a random variable is added for comparison (red vertical line). *P*, annual precipitation; Pvar, monthly precipitation variance; SWE, annual snow water equivalent; PET, annual potential evapotranspiration; BA, burned area; LC, land cover; subscripts 1 and 2, pre-fire and post-fire periods, respectively.

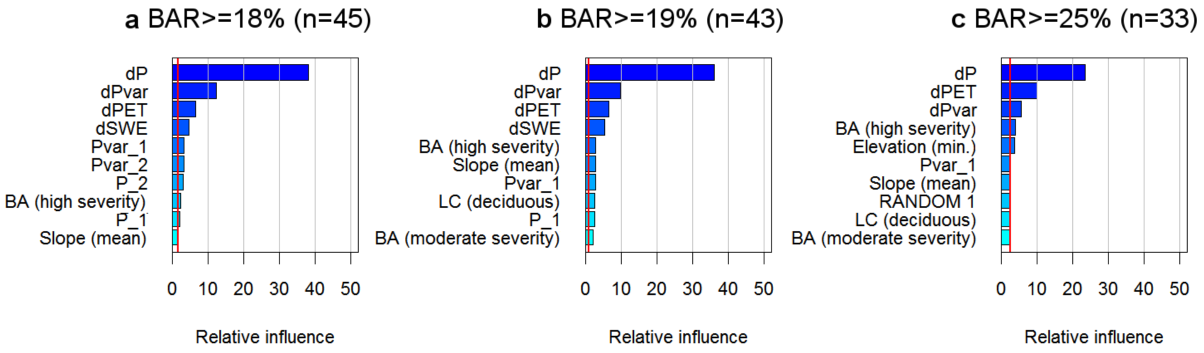

Supplementary Figure 2. Detection of the burn area to drainage area threshold with an impact on annual river flow in CONUS watersheds. Results show the relative influence of the ten most important variables on 5-year post-fire change in annual river flow for burn area to drainage area ratio (BAR) thresholds of 18% (**a**), 19% (**b**), and 25% (**c**). Influence is expressed as percentage of total influence of all variables, and was calculated based on the improvement in performance of the boosted regression model after inclusion of displayed variables. The relative influence of a random variable is added for comparison (red vertical line). *P*, annual precipitation; Pvar, monthly precipitation variance; SWE, annual snow water equivalent; PET, annual potential evapotranspiration; BA, burned area; LC, land cover; subscripts 1 and 2, pre-fire and post-fire periods, respectively.

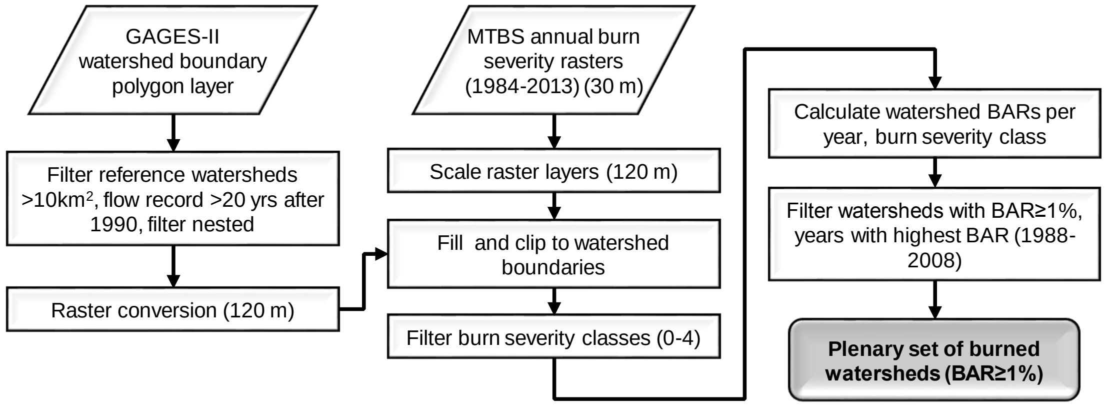

Supplementary Figure 3. Building the burned watershed database. BAR, burn area to drainage area ratio; GAGES, Geospatial Attributes of Gages for Evaluating Streamflow; MTBS, Monitoring Trends in Burn Severity.

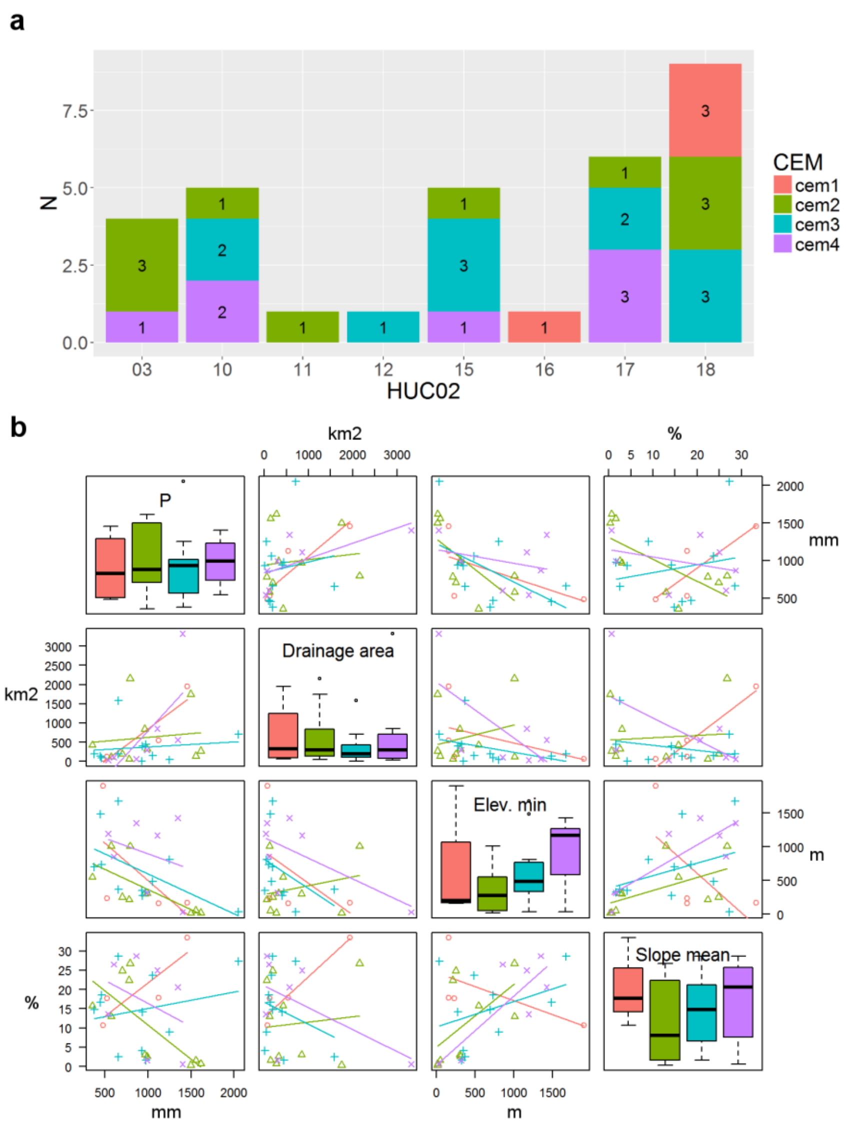

Supplementary Figure 4. Selection of climate elasticity models summarized by water resource region. (**a**) Best fitting climate elasticity models (CEM) (*BAR*≥19%) (*n*=32) and (**b**) corresponding values for pre-wildland fire precipitation *P*, drainage area, minimum elevation and mean slope per watersheds, summarized by CEM (*n*=32). The box-and-whisker plots along the diagonal show the univariate distributions grouped by CEM, with plot order indicated in the legend. Whiskers extend to the most extreme value no more than 1.5 × interquartile range from the box. The water resource regions (HUC-2) shown are: (3) South Atlantic-Gulf, (10) Missouri, (11) Arkansas-White-Red, (12) Texas-Gulf, (15) Lower Colorado, (16) Great Basin, (17) Pacific Northwest, and (18) California.

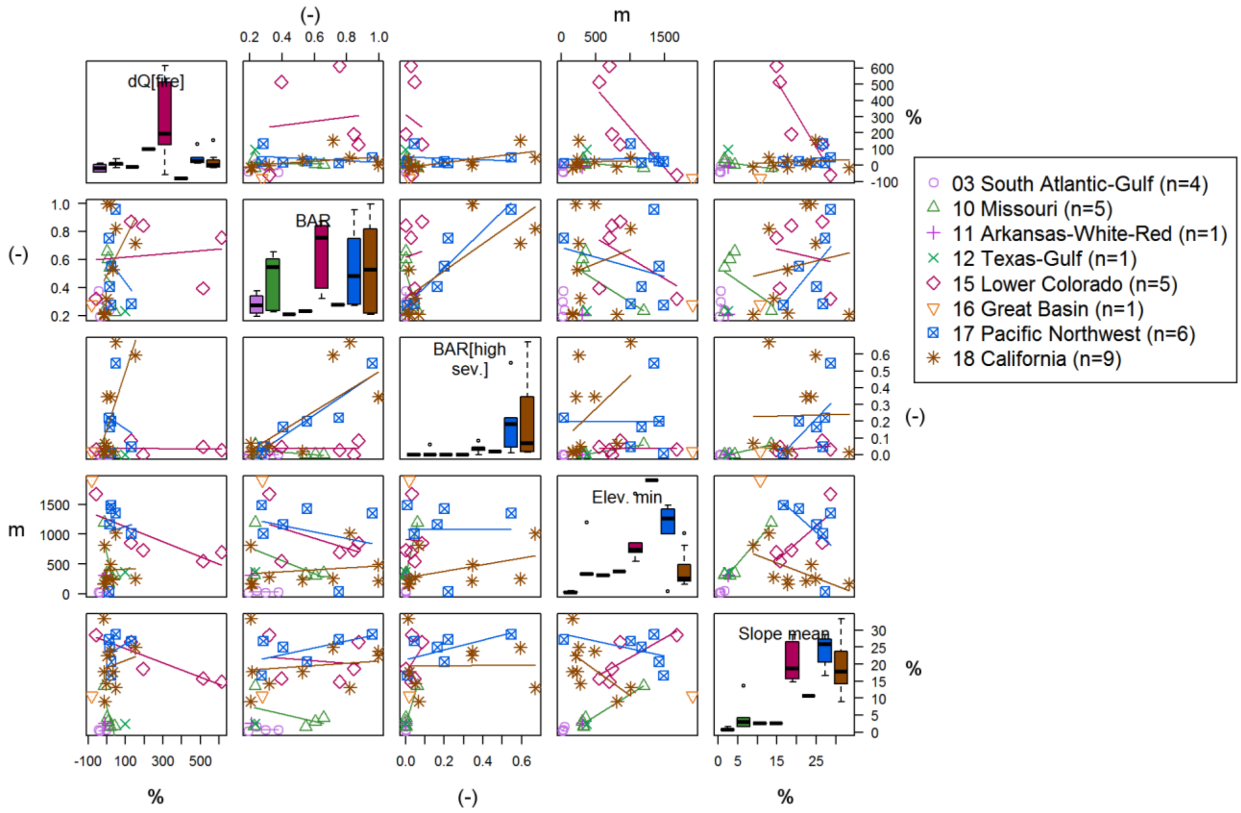

Supplementary Figure 5. Results of the attribution analysis summarized by water resource region. Multivariate relationships between changes in 5-year mean annual river flow *dQ* attributed to wildland fire, burned area ratio, and burned area ratio severely affected by fire in the period 1985–2008, and mean slope. Data points represent burned watersheds with BAR≥19% (*n*=32). The box-and-whisker plots along the diagonal show the univariate distributions grouped by water resource region, with plot order indicated in the legend. Whiskers extend to the most extreme value no more than 1.5 × interquartile range from the box. Scales are drawn along the vertical axes on either side of the plot.

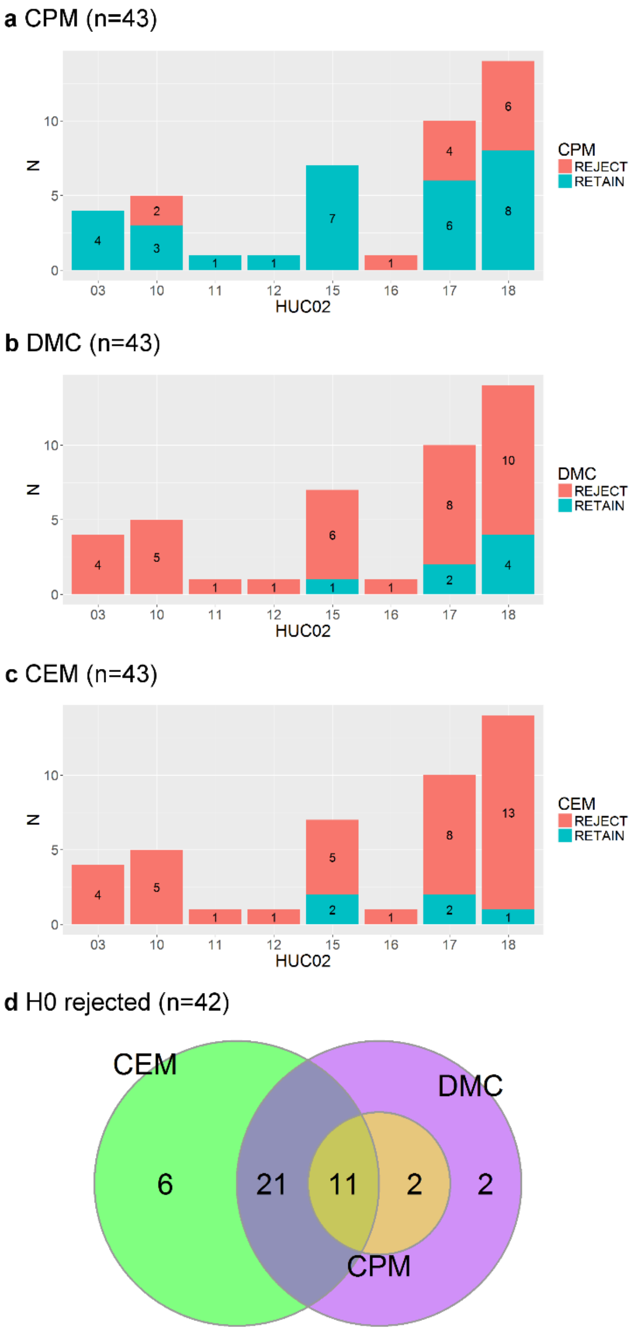

Supplementary Figure 6. Results of hypothesis testing. Results are shown for burned CONUS watersheds with BAR≥19% (*n*=43). Number of burned watersheds are shown per outcome ($p<0.05$) evaluated for the hypotheses of (**a**) no change in monthly river flow (*Q*) obtained with the CPM, (**b**) no change in monthly water yield ratio (*Q/P*) obtained with the DMC, (**c**) significance of the CEM, and (**d**) Venn diagram showing the number of burned watersheds with significant outcomes for all three tests. The water resource regions (HUC-2) shown are: (3) South Atlantic-Gulf, (10) Missouri, (11) Arkansas-White-Red, (12) Texas-Gulf, (15) Lower Colorado, (16) Great Basin, (17) Pacific Northwest, and (18) California.

Supplementary tables

Supplementary Table 1. Observed unattributed change in post-wildland fire annual river flow in the CONUS. Shown are the changes in 5-year mean river flow (*dQ*), summarized as median values in water resource regions (HUC-2) calculated for watersheds affected by wildland fire between 1985 and 2008 (BAR≥1%). Also shown are regional median values of drainage area, ratio of area burned to drainage area, and changes in 5-year mean precipitation (*dP*). *n* is the number of burned watersheds.

HUC-2 code	Water resource region	*n*	Drainage area (km^2)	BAR (%)	BAR_{ul} (%)	BAR_{mh} (%)	*dQ* (mm)	*dQ* (%)	*dP* (mm)	*dP* (%)
02	Mid Atlantic	4	547	3.1	2.6	0.5	-97.4	-16.5	-59.5	-4.3
03	South Atlantic-Gulf	37	413	3.1	2.5	0.1	-44.0	-12.9	-22.2	-1.3
04	Great Lakes	1	1910	1.8	0.3	1.5	-20.5	-5.1	-19.1	-2
05	Ohio	4	589	8.9	8.7	0.1	54.0	12.8	92.3	7.1
06	Tennessee	2	456	1.8	1.7	0.1	-69.0	-11.6	-76.6	-4.9
08	Lower Mississippi	3	342	4.8	4.3	0.2	160.1	27.4	311.2	23.3
09	Souris-Red-Rainy	1	2358	1.1	0.5	0.6	47.0	21.2	43.9	6.2
10	Missouri	15	471	6.0	2.9	0.2	-3.2	-5.3	20.3	2.1
11	Arkansas-White-Red	13	1820	4.2	4.2	0	1.4	16.0	53.7	4.6
12	Texas-Gulf	8	435	2.3	1.3	0.3	-10.7	-25.4	-26.6	-0.8
13	Rio Grande	3	163	4.5	1.4	3.0	-15.6	-29.8	-100.8	-13.1
14	Upper Colorado	3	333	2.5	1.3	2.2	77.5	19.8	41.9	6.9
15	Lower Colorado	15	425	15.4	9.3	3.8	9.9	25.6	-30.6	-6.0
16	Great Basin	5	70	5.8	5.3	0.9	-45.4	-37.1	0.7	0.1
17	Pacific Northwest	25	529	13.5	10.4	3.7	21.3	5.6	-28.5	-3.3
18	California	29	243	20.6	9.9	4.6	-38.4	-18.4	-35.4	-4.6
	CONUS	168	404	5.8	4.7	0.7	-5.9	-5.7	-23.1	-2.3

Abbreviations: BAR, burned area to drainage area ratio; BAR_{ul}, burned area to drainage area ratio for underburned to low burn severity; BAR_{mh}, burned area to drainage area ratio for moderate to high burn severity; CONUS, contiguous U.S.; HUC, hydrologic unit code (U.S. Geological Survey)

Supplementary Table 2. Climate elasticity models of river flow used in the attribution analysis. α and β are model coefficients. CEM, climate elasticity model; Q, river flow; P, precipitation; PET, potential evapotranspiration; $\sigma_{P_m}^2$, monthly precipitation variance; SWE, snow water equivalent.

Climate elasticity model	Equation	Significance test
CEM_0	$\frac{\mathrm{d}Q}{\overline{Q_0}} = 0$	(Null model)
CEM_1	$\frac{\mathrm{d}Q}{\overline{Q_0}} = \alpha \frac{\mathrm{d}P}{\overline{P_0}}$	*t*-test
CEM_2	$\frac{\mathrm{d}Q}{\overline{Q_0}} = \alpha \frac{\mathrm{d}P}{\overline{P_0}} + \beta \frac{dPET}{\overline{PET_0}}$	*F*-test
CEM_3	$\frac{\mathrm{d}Q}{\overline{Q_0}} = \alpha \frac{\mathrm{d}P}{\overline{P_0}} + \beta \frac{d\sigma_{P_m}^2}{\sigma_{P_{m,0}}^2}$	*F*-test
CEM_4	$\frac{\mathrm{d}Q}{\overline{Q_0}} = \alpha \frac{\mathrm{d}(P - SWE)}{(\overline{P_0} - \overline{SWE_0})} + \beta \frac{dSWE}{\overline{SWE_0}}$	*F*-test
